# On Modern Progress in Ophthalmic Medicine and Surgery

**Published:** 1893-11-25

**Authors:** Robert Brudenell Carter

**Affiliations:** Consulting Ophthalmic Surgeon to St. George's Hospital


### On Modern Progress in Ophthalmic Medicine and Surgery.

By Robert Brudenell Carter, F.R.C.S., Consulting Ophthalmic Surgeon to St. George's Hospital.

I mentioned in the preceding paper that the darkest period in the history of my subject was that which immediately preceded the dawn; and I cannot better display my conviction of the density of the darkness than by repeating part of the substance, and sometimes even the words, of an article which I wrote in 1865. "Twenty years ago," I then said, "ophthalmology would have furnished an admirable illustration of some of the positions laid down in South's well-known sermon, 'On the Terrible Imposture and Force of Words.'" The then and previously existing oculists, partly because they were, in fact, quite outside of any real knowledge of the diseases of the organ which they professed to treat, and partly through the tendency of mankind to surround a profitable monopoly by a defensive barrier, had exercised their ingenuity in the invention of ill-sounding and many-syllabled compounds, analogous to the Greek of the advertising tradesman, and intended to denote unreal or frivolous distinctions, which derived their sole importance from the jargon in which they were expressed. The "Eumetableton" of the carriage builder, or the "Antitribospodothecidion" of the upholsterer, were fairly rivalled by the classical learning which described a small protrusion of iris through a perforated cornea as "Myocephalon," by reason of its fancied resemblance to a fly's head; or by that which became responsible for "Amphiblestroiditis" as a designation of inflammation of the retina, without regard to the fact that "Amphiblestron" is not the equivalent of *rete*, but denotes specifically a casting net. A treatise on ophthalmic surgery, in those days, naturally concluded with a "Glossary," like that of a narrative of a sojourn among gipsies, or of a collection of ballads in a provincial dialect. The crown of glory in the art of word-making is fairly due to a German sciolist, who probably fancied himself a Porson or a Bentley, and who, having devised a small modification of the common procedure for overcoming obstruction of the lachrymal passages, conferred upon his method the title of "Dacryocystosyringokatakleisis." The bad writers of the present day, who are guilty of nothing worse than "Ophthalmoplegia," may well hide their diminished heads in the presence of this transcendant achievement.

The policy of the rulers of the profession, by whom the conditions of entrance within its portals were controlled, had then for some years been consistently in favour of a humble standard of general education among medical students. Why this was so it is not necessary here to inquire; but the fact remains. Among those who listened, as I did, to half-a-dozen lectures upon diseases of the eye, introduced, between fractures and herniæ, into the middle of the course upon general surgery, comparatively few would know the derivations, and fewer still would understand the supposed meanings, of the many long words which were employed. The majority, even if they kept awake, felt their energies give way beneath the burden of strange and incomprehensible sounds. They turned from the consideration of ophthalmic diseases partly on this account, and partly because their permitted ignorance had a still deeper and more important influence. It led to a common ambition among them to become "practical men." The ideal practical man was one who despised, and resisted the acquirement of, any kind of knowledge which did not, to his own immediate perception, visibly and directly lead him to the cure of disease. To such an one, whose higher intellectual faculties had been but faintly aroused by real mental training, the presence of doubt and difficulty was nothing more than a source of annoyance. The men of this class could set a fractured tibia, or reduce a dislocated femur. The best of them could even administer mercury and use belladonna in a case of manifest iritis. But with regard to the more obscure sources of blindness, they saw no harm in confessing themselves ignorant, and they certainly had very few opportunities of exchanging their ignorance knowledge.

Almost contemporaneously with the invention of the ophthalmoscope the late Professor Donders, of Utrecht, applied himself to the study of the defects of sight which were dependent upon faulty shape of the eyeball or upon deficiency or impairment of its power of adjusting itself for different distances, and which were capable of correction and relief by means of optical appliances. Questions of this kind had previously been left in the hands of so-called "opticians," who, even if they were capable of understanding the physical conditions involved, had no acquaintance with either the physiology of the eye or with its diseases, and were therefore unable to allow for two of the most important factors in many of the cases which were presented to them. In consequence of this want of knowledge they fell into errors which still survive in what is called the public mind, and which are every now and then advanced by patients, who display quite a touching confidence in them as rules of conduct which it is proper to ob serve. To this branch of the subject Mr. Zachariah Laurence gave especial attention, and his work on the optical defects of the eye and their consequences did more to advance and extend a knowledge of these conditions in England than even the translation of the works of Donders, published in 1864 by the New Sydenham Society. In the same year Mr. Laurence established the *Ophthalmic Review*, the first English journal devoted to this branch of practice. It gave publicity to the best work which was being done in the department in other countries; and, at the same time, became somewhat of a terror to careless or ignorant authors. There was a general impression that discoveries were being made in relation to ophthalmic work; and some were eager to represent themselves as possessors of the new knowledge before, as a matter of fact, they had taken the pains necessary to acquire it. Upon such Mr. Laurence and his coadjutors had no mercy; and in this way the *Review* did good service in establishing and maintaining a high standard of literary work. At the same time the late Sir William Bowman and the late Mr. Critchett, at the Royal London Ophthalmic Hospital, were bringing open minds to bear upon every real or supposed improvement, and, notwithstanding some opposition from less enlightened persons, were testing the various suggestions which reached them from every quarter. To some of these, in their more immediately useful aspects, I shall hope to call attention in my next.

